# The ameliorative effects of hesperidin in rats developed hepatotoxicity with deltamethrin

**DOI:** 10.22038/ijbms.2025.82598.17854

**Published:** 2025

**Authors:** Seda Cetinkaya Karabekir, Mehmet Enes Sozen, Ilknur Cinar Ayan, Hasan Basri Savas, Gokhan Cuce, Serpil Kalkan

**Affiliations:** 1 Izmir Bakırcay University, Faculty of Medicine, Department of Histology and Embryology, Izmir, Turkey; 2 Alanya Alaaddin Keykubat University, Faculty of Medicine, Department of Histology and Embryology, Alanya, Turkey; 3 Necmettin Erbakan University, Faculty of Medicine, Department of Medical Biology, Meram, Konya, Turkey; 4 Mardin Artuklu University, Faculty of Medicine, Department of Medical Biochemistry, Mardin, Turkey; 5 Necmettin Erbakan University, Faculty of Medicine, Departments of Histology and Embryology, Meram, Konya, Turkey

**Keywords:** Classical Apoptosis, Hesperetin- 7-Rhamnoglucoside, Insecticides, Oxidative Stresses, Toxicity

## Abstract

**Objective(s):**

Deltamethrin (DLM) is a widely used insecticide in agriculture; however, exposure to it can lead to serious health problems. This study aimed to evaluate the protective effects of hesperidin (HSP), a natural antioxidant, against DLM-induced liver toxicity.

**Materials and Methods:**

Thirty-two male Wistar Albino rats (250–300 g, 4 months old) were divided into four groups. The control group received 1 ml of corn oil via oral gavage for 30 days. The DLM group received 1.28 mg/kg DLM in corn oil for 30 days. The DLM+HSP 100 mg/kg and DLM+HSP 300 mg/kg groups received 1.28 mg/kg DLM followed by 100 mg/kg or 300 mg/kg HSP in distilled water, respectively, 30 min after DLM administration for 30 days. Liver tissues were examined histopathologically. Masson’s trichrome staining and PCR assessed fibrosis. Caspase 3 and 9 expressions in liver tissues were determined by immunohistochemistry and PCR. Biochemical analyses were conducted on serum samples.

**Results:**

HSP supplementation led to a dose-dependent decrease in aspartate aminotransferase (AST) and alanine aminotransferase (ALT) levels. DLM exposure decreased antioxidant capacity, while HSP supplementation increased it dose-dependently. Histopathological evaluations showed increased liver damage in the DLM group, while HSP administration reduced liver toxicity. Masson’s trichrome staining and analysis of collagen I (COL1A1) and collagen III (COL3A1) gene expression revealed increased fibrosis in the DLM group, which was attenuated with HSP treatment.

**Conclusion:**

The potential prevention of DLM-induced liver toxicity and apoptosis by HSP may be an alternative protective strategy.

## Introduction

Insecticides have been utilized in agriculture for an extended period, significantly contributing to increased agricultural productivity (1). Deltamethrin (DLM) (S)-alpha-cyano-3-phenoxybenzyl-(1R)-cis-3-(2,2-dibromovinyl)-2, 2-dimethylcyclopropane carboxylate is a Type II synthetic pyrethroid widely employed as an insecticide and acaricide globally (2) Recognized as one of the most potent insecticides, it is extensively applied across terrestrial and aquaculture industries, including agriculture, fruit and vegetable farming, and fisheries, to combat pests and parasites such as lice, flies, and ticks (3-7). It is frequently chosen because of its quick metabolism and minimal toxicity to mammals when exposed (8). However, recent studies on the toxicity effects of DLM and its metabolites have demonstrated that these substances exhibit multi-organ toxicity, which can lead to reproductive and developmental toxicity, endocrine-disrupting toxicity, and liver toxicity, among other effects (9-11). In addition to its other effects, DLM has been shown to produce reactive oxygen species (ROS) and induce oxidative stress, which can result in lipid peroxidation, DNA damage, and protein denaturation (12). The possible health effects of pesticides on humans, including acute, subacute, and chronic poisoning, have drawn attention to their extensive use. The liver is the primary site of drug metabolism and is among the tissues most frequently affected by chemicals. Recent studies have shown that pesticide exposure, including DLM, is associated with the onset and progression of liver diseases (13, 14). The accumulation of DLM, especially in liver tissue, increases the formation of ROS and damages proteins, nucleic acids, and lipids. Therefore, the search for ways to protect against liver toxicity has attracted the attention of many researchers. Natural antioxidants that can reduce DLM-induced tissue damage are thought to be useful for this purpose (15, 16).

Plant phenolics known as flavonoids have low molecular weights and flavan nuclei. Plants generate flavonoids, which are hydroxylated phenolic compounds with well-studied antioxidant effects that protect against microbial infections (17).

HSP (3,5,7-trihydroxy flavanone-7-rhamnoglucoside) is a bioflavonoid abundant in citrus fruits, including orange, lemon, and grapefruit, and has active pharmacological activities. HSP is capable of scavenging hydrogen peroxide and hydrogen radicals. Lipid-lowering, anti-inflammatory, antioxidant, anti-hypertensive, anti-carcinogenic, and anti-edema activities have been reported for HSP (18). In addition, HSP has beneficial pharmacological and biological properties such as antiallergic, antibacterial, antiviral, and neuroprotective (19). Based on numerous research articles, these properties have been reported to demonstrate that hesperidin protects against liver damage induced by natural and chemical toxins mediated by inflammation and/or oxidative stress (20).

Many structures are involved in the antioxidant system that neutralizes oxidative products. These parameters should not be evaluated in a unidirectional manner since the situation will be tried to be balanced by increasing antioxidant activity as a reactive response to increased oxidative stress. Parameters indicating both oxidative stress level and antioxidant level should be evaluated together. It is known that free radicals formed due to disruption of the balance between oxidative stress/antioxidant status cause more than 100 serious diseases. Many diseases occur if the antioxidant system cannot compensate for increased oxidative stress (21-24).

Thiol balance is a new biochemical marker for antioxidant capacity and oxidative stress assessment. These markers are studied from serum obtained by centrifugation of blood in a biochemistry tube (gel). Because they are new, they have high specific values. They are evaluated for many disease groups and are not sufficiently included in the literature. Research with these markers will contribute to the literature (16, 25-27).

This study investigated the possible protective effect of HSP against DLM-induced oxidative stress and hepatotoxicity at different doses by histologic, biochemical, and molecular methods.

## Materials and Methods

The Necmettin Erbakan University Animal Experiments Local Ethics Committee approved the study with a decision dated 05.11.2021 and numbered 2021-052. 

### Animals and experimental groups

In our study, 32 male Wistar Albino rats (4 months old, weighing 250-300 g) were divided into four groups, with eight rats in each group. Control group: 1 ml of corn oil was administered by oral gavage for 30 days. DLM group: 1,28 mg/kg DLM was dissolved in 1 ml of corn oil and given by oral gavage for 30 days. DLM+HSP 100 mg/kg: After the administration of DLM (1,28 mg/kg) in 1 ml of corn oil, 100 mg/kg of Hesperidin (HSP) was dissolved in distilled water and administered by gavage 30 min later. DLM+HSP 300 mg/kg: After the administration of DLM (1,28 mg/kg) in 1 ml of corn oil, 300 mg/kg of Hesperidin (HSP) was dissolved in distilled water and administered by gavage 30 min later.

DLM (45423 Supelco® CAS No: 52918-63-5) and HSP (CAS No: 520-26-3 Sigma Aldrich HSP) were freshly prepared before each application.

The oral LD50 value for DLM rats is 128 mg/kg (28). The selected DLM dose was based on the previous study in which 1.28 mg/kg, corresponding to 1/100 of the LD50, caused biochemical and histopathological changes in rats. 

According to Li *et al*. HSP’s acute and subchronic oral toxicity was analyzed with urine analysis, hematology, clinical biochemistry, organ weights, pathology, and food consumption were all normal in the subchronic toxicity study when HSP (250 and 500 mg/kg) was used; however, changes in body and organ weights, hematology, tissue histopathology, and clinical biochemistry were significantly (*P*<0.05) altered when HSP (1000 mg/kg) was used. In summary, experiments on male and female rats at 1000 mg/kg have shown that HSP has few side effects (29). For this reason, our study chose doses of 100 mg/kg and 300 mg/kg for HSP.

The rats’ weights were measured both before and after the experiment, and the results were noted. At the end of 30 days, blood samples and liver tissue were collected under anesthesia with 50 mg/kg Ketamine HCl + 10 mg/kg Xylazine HCl injection, and cervical dislocation was performed. 

### Biochemical analyses

Blood samples from each experimental animal were gathered afterward, placed in a biochemistry tube with gel, and centrifuged at 1500 g for ten minutes to extract the supernatant serum fraction. The serum samples were then portioned and labeled in Eppendorf tubes and kept at -80 ^°^C until analysis. At the time of the study, all serum samples were thawed simultaneously and brought to room temperature. Then, all serum samples were mixed with the help of a vortex device and prepared for biochemical analysis. 

Serum thiol-disulfide balance was determined by a newly developed manual colorimetric method using a commercial kit. The measurement method employed in this study is a widely recognized colorimetric technique that has been extensively documented and utilized in prior scholarly works. Reducible disulfide bonds were first reduced to form free functional thiol groups. The unused reductant sodium borohydride was consumed and removed with formaldehyde, and after reaction with DTNB (5, 5-dithibis-2-nitrobenzoic acid), all thiol groups, including reduced and native thiol groups, were determined. Half the difference between total and native thiol gives the dynamic disulfide amount. After determining native thiol and disulfide amounts, total thiol amount, native thiol/total thiol ratio, disulfide/total thiol ratio, and disulfide/native thiol ratio were calculated. In addition, routine biochemical parameters, especially AST and ALT levels, were studied colorimetrically on an autoanalyzer (25-27)

### Histological examinations

After blood samples were collected, the abdominal cavities of the sacrificed rats were opened, and liver tissues were fixed in 10% buffered neutral formalin following macroscopic examination. After tissue processing, 5 µm-thick sections were randomly selected from paraffin blocks of each liver tissue sample for microscopic analysis. The sections were deparaffinized in xylene (3 x 20 min) and processed through a graded alcohol series (100%, 90%, 80%, 70%, and 50%). Fibrosis evaluation was performed using the Masson-Trichrome staining method. Additionally, histopathological scoring based on parameters such as sinusoidal dilatation, congestion, pyknotic nuclei, and mononuclear cell infiltration was conducted using Hematoxylin and Eosin (H&E) staining. Histopathological changes were graded as absent (0), mild (1), moderate (2), and severe (3) (30). Following the staining procedures, the sections were dehydrated using a graded alcohol series and finalized with a mounting medium. Histopathological examination was performed in six different areas of each section using a double-blind method. The evaluations were conducted using a Zeiss Lab.A1 light microscope and a Zeiss Axicam ERc 5s camera.

### Immunohistochemistry

Caspase 3 (ab184787) and caspase 9 (ab210611) antibodies were used for immunohistochemistry as primer antibodies. Paraffin was removed from 5 μm sections using xylene for 30 min. Sections were placed in Super Block (ScyTek Laboratories, Logan, UT) for 10 min, then washed with PBS for 5 min, incubated with primary antibody for 60 min, washed with PBS for 5 min, then incubated with secondary antibody for 20 min, and washed again with PBS for 5 min. Then, Streptavidin-peroxidase was added and incubated for 20 min, then washed with PBS for 5 min. AEC (Sigma Alderich AEC101-1KT) chromogen was added and incubated for 15 min, then washed with distilled water for five minutes. Mayer’s hematoxylin was used as a background stain after allowing the visible immune reaction to occur under the microscope. Sections covered using a water-based covering medium were examined under a Zeiss Lab.A1 light microscope and evaluated with a Zeiss Axicam ERc 5s camera imaging system. At the end of the evaluations, immunohistochemistry staining for each primary antibody was assessed according to the following criteria: 0, no staining; 1, weak staining; 2, moderate staining; and 3, intense staining (31).

### RNA extraction and real-time qPCR

RT-qPCR analysis was performed to determine the mRNA levels of CASP3, CASP9, COL1A1, and COL3A1 genes related to apoptotic cell death pathways and collagen synthesis. Total RNA of liver tissue samples from the control and dose group (DLM, DLM+HSP 100, DLM+HSP 300) were extracted using RiboEX total RNA isolation reagent (GeneAll, 301-001). DNase I enzyme was used to remove possible DNA contamination from the total RNA obtained from each sample (Thermo Fisher Scientific, #EN0521). Following enzyme treatment, cDNA was synthesized from 1 μg of total RNA with the iScript cDNA Synthesis kit (Bio-Rad) (Bio-Rad, 170-8891). Primers for the following target genes and GAPDH reference gene used in RT-qPCR analysis were designed with IDT PrimerQuest (https://eu.idtdna.com/Primerquest/Home/Index) online program: CASP3 forward, 5′- CCC TGA AAT GGG CTT GTG TA -3′ and reverse, 5′- GAG GTT AGC TGC ATC GAC AT -3′; CASP9 forward, 5′- GAA GAA CGA CCT GAC TGC TAA G -3′ and reverse, 5′- ATG AGA GAG GAT GAC CAC CA -3′; COL1A1 forward, 5′- CCA ATG GTG CTC CTG GTA TT -3′ and reverse, 5′- GTT CAC CAC TGT TGC CTT TG -3′; COL3A1 forward, 5′- GTG TGA TGA TGA TGA GCC ACT AGA C -3′ and reverse, 5′- TGA CAG GAG CAG GTG TAG AA -3′; GAPDH forward, 5′- GCA TTG CAG AGG ATG GTA GAG -3′ and reverse, 5′- GCG GGA GAA GAA AGT CAT GAT TAG -3′. For the RT-qPCR reaction, 5 µl 5x HOT FIREPol® EvaGreen® qPCR master Mix Plus (ROX) (Solis BioDyne), 5 pMol forward primer, 5 pMol reverse primer and 2 µl cDNA were used. The PCR protocol consisting of an initial activation step at 95 C0 for 12 min, 40 cycles of 95 C0 for 15 sec, 60 C0 for 20 sec, and 72 C0 for 20 sec amplification step was performed on Bio-Rad CFX ConnectTM Real-Time System.

### Statistical analysis

**Statistical** **analysis** **and** **calculations** **were** **performed** **in** **GraphPad** **Prism 8.**

Histologic changes and biochemical results were expressed as mean and standard deviation. Shapiro-Wilk and One-Sample Kolmogorov-Smirnov were used to test the conformity of the data of the groups to normal distribution. It was determined that all groups showed normal distribution. A one-way analysis of variance (ANOVA) in independent groups and a parametric test was applied to the study groups. *Post-hoc* Tukey’s HSD test was preferred for pairwise comparisons after ANOVA. Immunohistochemical data not showing normal distribution were compared using the Kruskal-Wallis test, and differences between paired groups were compared using Dunn’s test. Statistical analysis of the differences in the mRNA level of each gene between the groups was performed by the 2^–ΔΔCT^ method. A significance value of *P*<0.05 was accepted.

## Results

### Hematoxylin and eosin staining

**Figure 1:**
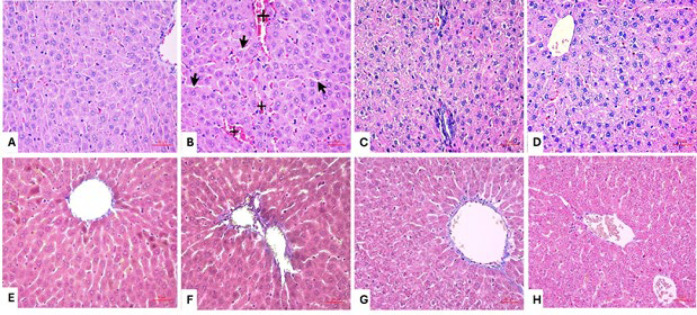
H&E staining of rat liver section specimens A; Control, B; DLM C; DLM+HSP100, D; DLM+HSP300 black arrow; dilatation, crosshair; congestion. Masson staining of liver sections E; Control, F; DLM, G; DLM+HSP100, H; DLM+HSP300. H&E and Masson Staining; scale bars=50 µm

**Figure 2:**
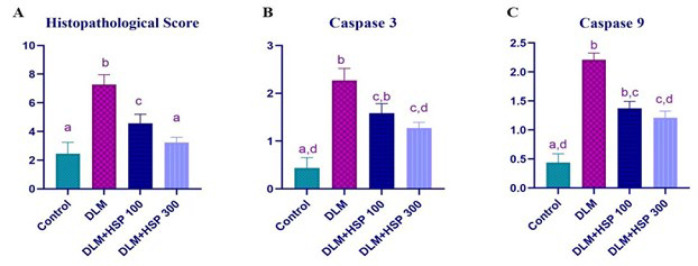
(A) Preparations were evaluated for sinusoid dilatation, congestion, pyknotic nuclei, and mononuclear cell infiltration. Histopathologic changes in rat livers were graded as absent (0), mild (1), moderate (2) and severe (3). The maximum score was determined as 12. Oneway ANOVA was evaluated with the Tukey Test (*P<*0.05). a, b, c indicates statistical significance between groups (*P˂*0.05). (B) Caspase 3 and (C) Caspase 9 expression immunohistochemical scoring a, b, c, and d indicate statistical significance between groups (*P˂*0.05)

Histological examination revealed normal liver structures in the control group. In contrast, the DLM group exhibited changes such as sinusoidal dilatation, congestion, pyknotic nuclei, and mononuclear cell infiltration (Figure 1). Histopathologic changes were graded as absent (0), mild (1), moderate (2), and severe (3)(30). Hesperidin (HSP) administration improved DLM-induced liver damage in a dose-dependent manner (Figure 2A). Histopathological findings in the DLM and DLM+HSP 100 groups were significantly higher compared to the control group (*P*<0.0001). In contrast, no significant difference was observed between the DLM+HSP 300 and the control groups (*P*=0.0989). The DLM group showed significantly more damage than the DLM+HSP 100 and 300 groups (*P*<0.0001). A significant difference was found between the DLM+HSP 100 and 300 groups, highlighting the liver-healing effects of high-dose HSP (*P*=0.0012).

### Masson staining

Histological changes were not detected in the control group, and no increase in fibrosis was observed. The DLM group observed a higher degree of fibrosis in the portal area and its surrounding regions than the control group. A dose-dependent decrease in fibrosis was found in the groups treated with HSP compared to the group treated with DLM (Figure 1). 

### Immunohistochemistry

**Figure 3:**
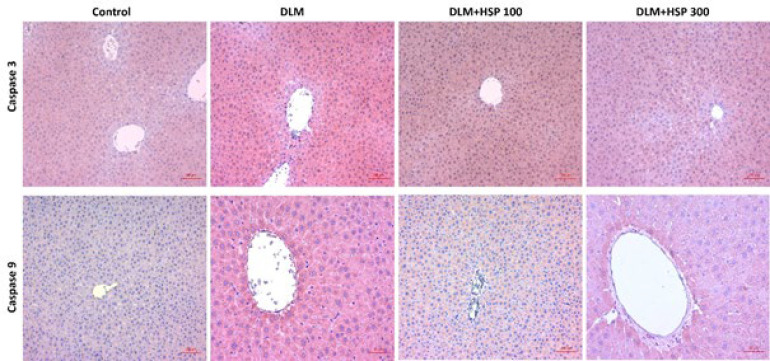
Immunohistochemical staining images of caspase 3 and caspase 9 expressions in liver tissues of all rat groups

DLM administration significantly increased Caspase-3 and Caspase-9 expression, while HSP 100 and HSP 300 applications modulated this effect dose-dependently. Regarding Caspase-3 expression, the DLM group showed a significant increase compared to the Control group (*P*<0.0001). The difference between the Control and DLM+HSP 100 groups was statistically significant (*P*=0.0065), and similarly, a significant difference was observed between the DLM and DLM+HSP 300 groups (*P*=0.0065)(Figure 2 B, Figure 3). 

A similar trend was noted in Caspase-9 expression, where DLM administration significantly increased compared to the Control group (*P*<0.0001). The difference between the Control and DLM+HSP 100 groups was statistically significant (*P*=0.0098), and a significant difference was also found between the DLM and DLM+HSP 300 groups (*P*=0.0098). These findings suggest that DLM promotes apoptotic mechanisms, while HSP 300 suppresses this effect (Figure 2C, Figure 3).

### mRNA expression analysis of apoptosis and collagen synthesis-related genes

**Figure 4:**
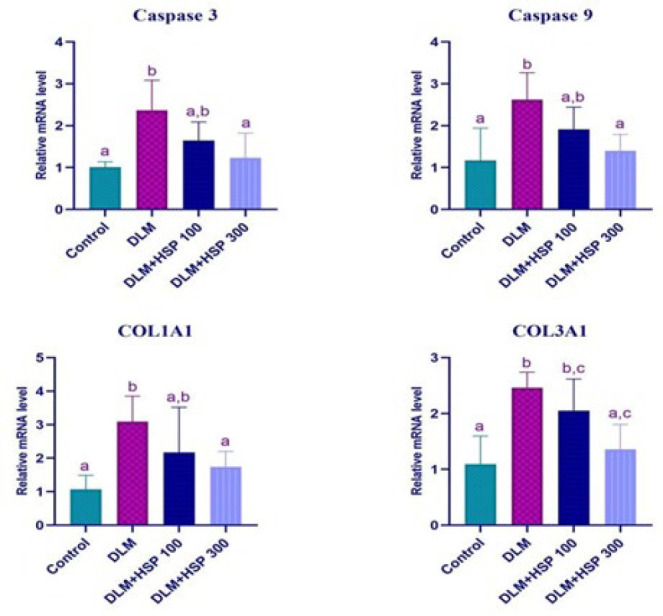
mRNA expression analysis of Caspase 3, Caspase 9, COL1A1, and COL3A1 genes a, b, and c indicate statistical significance between groups (*P˂*0.05)

The effects of DLM and different doses of HSP on apoptosis and collagen synthesis in liver tissues from the experimental dose groups were evaluated at the molecular level by RT-qPCR analysis. According to the results of this analysis, Caspase 3 mRNA expression increased 2.35-fold (*P*=0.001) in tissues treated with DLM alone compared to the control group. Caspase 3 gene expression decreased 1.44-fold (p=0.1066) and 1.92-fold (*P*=0.0056) in DLM+HSP 100 and DLM+HSP 300 treated groups compared to the DLM alone treated group (Figure 4).

When the change in the mRNA expression of Caspase 9 in the dose groups compared to the control group was evaluated, it was determined that it increased 2.24-fold (*P*=0.0024) only in the DLM-treated group. When compared to the DLM alone treatment group, it decreased 1.37-fold (*P*=0.2064) and 1.88-fold (*P*=0.0105) in DLM+HSP 100 and DLM+HSP 300 treated groups, respectively (Figure 4).

When the mRNA expression level results of the Col1A1 gene were analyzed, it was found that it increased 2.89-fold (*P*=0.0023) in the DLM alone treatment group compared to the control group, while it decreased 1.42-fold (p=0.2554) and 1.78-fold (*P*=0.0473) in the DLM+HSP 100 and DLM+HSP 300 treated groups compared to the DLM alone treated group, respectively (Figure 4).

When Col3A1 mRNA gene expression results were evaluated, a 2.25-fold (*P*=0.0003) increase was determined in the DLM dose group compared to the control group. When compared to the group treated with DLM alone, a 1.2-fold (p=0.4319) and 1.81-fold (*P*=0.0026) decrease was detected in DLM+HSP 100 and DLM+HSP 300 groups, respectively (Figure 4).

### Biochemical analyses

Biochemical results were evaluated using the ANOVA test and *post-hoc* Tukey test. Administration of DLM significantly increased AST levels compared to the Control group (*P*=0.0001), as well as the DLM+HSP 100 and DLM+HSP 300 groups (*P*<0.0001). Similarly, ALT levels were significantly higher in the DLM group compared to the Control group (*P*=0.0004) and the DLM+HSP 100 and DLM+HSP 300 groups (*P*<0.0001). The AST/ALT ratio also showed a significant increase in the DLM group compared to the Control (*P*=0.0089), DLM+HSP 100 (*P*=0.0108), and DLM+HSP 300 (*P*=0.0016) groups.

Regarding oxidative stress markers, total thiol levels were significantly decreased in the DLM (*P*=0.0006) and DLM+HSP 100 (*P*=0.0037) groups compared to the Control group. Additionally, the DLM+HSP 300 group exhibited a significant increase in total thiol levels compared to the DLM group (*P*=0.0217). Native thiol levels were also significantly reduced in the DLM (*P*=0.0005) and DLM+HSP 100 (*P*=0.0015) groups compared to the Control group.

These findings suggest that DLM administration leads to hepatotoxic effects, as evidenced by increased liver enzyme activities and alterations in oxidative stress markers. The co-administration of HSP 100 did not mitigate these effects, whereas HSP 300 co-administration appeared to partially restore total thiol levels, indicating a potential protective role against DLM-induced oxidative stress.

## Discussion

The liver is the principal site of DLM toxicity due to its central role in DLM metabolism (32, 33). Metabolites accumulated in the target tissue cause damage to cells by causing ROS generation (34). Although several reports have been published on the toxicity of DLM, little work has been done on using natural products to prevent such toxicity and the mechanism of their ameliorative effects (35).

Plants are a significant source of biologically active compounds that protect the environment, keep people safe, and preserve biodiversity. Because of their antioxidant properties, these bioactive chemicals have several positive benefits for cancer, inflammation, and cardiovascular disease. Natural antioxidants are also essential in preventing hepatotoxicity caused by xenobiotics such as pesticides (36). We investigated the protective capacity of HSP against DLM-induced hepatotoxicity.

Abdel-Daim *et al*. reported that DLM toxicity increased serum biomarkers of liver injury such as AST, ALT, alkaline phosphatase (ALP), low molecular weight lipoprotein (LDL), cholesterol, and total bilirubin and decreased serum protein levels. Although these indicators are not specific to liver damage, their high activity indicates that the liver is no longer functioning (35). A further investigation documented that elevated levels of serum AST and ALT activity in quail, caused by liver injury induced by a dosage of 45 mg/kg DLM over 12 weeks, were attributed to the loss of hepatocyte membrane integrity or necrosis (37). Yousef *et al*. administered 1.28 mg/kg DLM as a single dose for 30 days in Sprague-Dawely rats. They reported that total lipid (TL), cholesterol, triglyceride (TG), LDL, and very low molecular weight lipoprotein (VLDL) increased significantly. In contrast, high molecular weight lipoprotein (HDL) levels decreased (38). In our study, by the other studies, a statistically significant increase was observed in AST and ALT markers related to liver damage in the DLM group. At the same time, HSP supplementation decreased in proportion to the dose. The dose-dependent reduction of these biochemical changes induced by DLM with hesperidin (HSP) supplementation is remarkable. Through its antioxidant and cytoprotective properties, hesperidin may have alleviated the oxidative stress and inflammatory responses caused by DLM. This suggests that hesperidin administration supports hepatocyte membrane stabilization, thereby reducing cellular damage.

In addition, a previous study (39) reported that superoxide dismutase (SOD), catalase (CAT), and glutathione peroxidase (GPx) activities decreased in the group administered 6.25 mg/kg DLM daily by gavage for 120 days. Another study reported that SOD, CAT reduced glutathione (GSH), and GPx levels decreased in the group given 15 mg/kg DLM for two weeks (36). Native Thiol and Total Thiol values are expected to increase with increased antioxidant capacity. Our study results show that DLM exposure resulted in a decrease in antioxidant capacity, according to our hypothesis regarding oxidant-antioxidant balance, and HSP supplementation resulted in an increase in antioxidant capacity in proportion to the dose. In addition, in line with our hypothesis, it can be evaluated that DLM exposure increases oxidative stress, and HSP supplementation leads to a decrease in oxidative stress in proportion to the dose. 

A study (37) suggested that 45 mg/kg DLM-induced liver damage was associated with steatosis and inflammation based on the results of H&E staining. Rjeibi *et al*. (36) reported that histopathological lesions, including membrane cell disruption, lipid vacuolization, and congestion, were observed in the livers of rats exposed to 15mg/kg DLM for 4 weeks. They reported that these changes observed in the general histology of the liver may be due to increased ROS production caused by the toxic effects of DLM (36). Shaheen and Ibrahim (40) investigated the effect of HSP against liver damage caused by Lambda Cyhalothrin (LCT), a pesticide. H&E stained liver sections of the LCT group hepatocytes showed degenerative nuclei vacuolized cytoplasm, and some hepatocytes showed pyknotic nuclei. It was also reported that congestion, inflammatory cell infiltration, enlarged portal vein, and bile duct hyperplasia were observed (40). Shaheen and Ibrahim (40) found that the LCT group had significantly more collagen fibers between hepatocytes and surrounding the portal triad. In addition, the LCT +HSP 100mg/kg group had significantly fewer collagen fibers between their hepatocytes. Li *et al*.(37) also showed that DLM exposure induced liver fibrosis in quail. Oxidative stress is the primary pathogenesis of liver fibrosis (41). Masson trichrome staining data of our study showed that DLM administration increased fibrosis in the liver. In contrast, HSP administration decreased the increase in fibrosis in proportion to the dose by other studies. Furthermore, it was observed that the DLM group exhibited a statistically significant upregulation in the expression of the COL1A1 and COL3A1 genes compared to the control group. The DLM+HSP 300 group exhibited a statistically significant reduction in COL1A1 and COL3A1 gene expression compared to the DLM group. HSP’s anti-inflammatory and antioxidant properties play an important role in liver fibrosis. HSP may inhibit collagen accumulation between hepatocytes by regulating pro-inflammatory cytokines and oxidative stress.

In a study (15), the group exposed to 6.75 mg chronic chlorpyrifos (CPF), had a higher caspase and Bcl-2 Associated X-protein (Bax) expression and decreased B-cell lymphoma 2 (Bcl-2) level than the control group. On the other hand, HSP administration was reported to decrease caspase 3 and Bax levels while increasing Bcl-2 levels. Thus, HSP reduced increased apoptosis caused by CPF in the liver (15). Shaheen and Ibrahim (40) reported that liver sections in the LCT group showed positive immunoreaction for iNOS and caspase 3 in the hepatocyte cytoplasm and near the central vein. In contrast, in the LCT+HSP 100 mg/kg group, several hepatocytes showed weak immunoreaction for iNOS and caspase 3 in the cytoplasm (40). Abdelaziz *et al*. (19) showed that using HSP with methotrexate (MTX) increased the expression of Nrf2 and HO-1 compared to the MTX group. HSP attenuated the inflammatory response by tumor necrosis factor α (TNF-α) together with a decrease in the nuclear factor-κB (NF-κB) pathway, indicating that it prevents MTX-induced liver inflammation. Similarly, the anti-apoptotic effect of HSP was demonstrated by decreased p53, increased Bcl-2, and significantly reduced caspase 9 mRNA expression compared to the MTX group (19). In our study, as a result of immunohistochemistry staining and PCR analysis, it was observed that caspase 3 and caspase 9 expression increased statistically significantly with DLM application, DLM+HSP 300 application decreased this increase statistically significantly and thus reduced DLM-induced apoptosis. These findings suggest that HSP can modulate the cellular death mechanisms of the liver and could potentially prevent DLM-induced hepatotoxicity. The ability of HSP to inhibit pro-apoptotic and pro-inflammatory signaling pathways can be associated with reducing oxidative stress and maintaining cellular homeostasis.

DLM; Deltamethrin, HSP; Hesperidin, H&E; Hematoxylin and Eosin

DLM; Deltamethrin, HSP; Hesperidin

DLM; Deltamethrin, HSP; Hesperidin

DLM; Deltamethrin, HSP; Hesperidin

## Conclusion

This study observed that DLM induces oxidative stress, hepatotoxicity, apoptosis, and fibrosis in the liver. Hesperidin (HSP) reduces these adverse effects of DLM in a dose-dependent manner, preventing oxidative stress, liver damage, and apoptosis. HSP’s antioxidant properties play an effective role in liver protection, making it a promising therapeutic option for preventing DLM-induced damage. 

## Data Availability

The data supporting this study’s findings are available upon request from the corresponding author.

## Declaration

We have not used AI tools or technologies to prepare this manuscript.
